# Prognostic Role of PD-L1 Expression in Invasive Breast Cancer: A Systematic Review and Meta-Analysis

**DOI:** 10.3390/cancers13236090

**Published:** 2021-12-03

**Authors:** Magno Belém Cirqueira, Carolina Rodrigues Mendonça, Matias Noll, Leonardo Ribeiro Soares, Maria Auxiliadora de Paula Carneiro Cysneiros, Regis Resende Paulinelli, Marise Amaral Rebouças Moreira, Ruffo Freitas-Junior

**Affiliations:** 1 Postgraduate Program in Health Sciences, Universidade Federal de Goiás, Goiás 74605-050, Brazil; carol_mendonca85@hotmail.com (C.R.M.); matias.noll@ifgoiano.edu.br (M.N.); nenacys@hotmail.com (M.A.d.P.C.C.); marisemoreira7@gmail.com (M.A.R.M.); 2 Instituto Federal Goiano (IF Goiano), Goiás 74270-040, Brazil; 3 Department of Sports Science and Clinical Biomechanics, University of Southern Denmark, 5230 Odense, Denmark; rrpaulinelli@gmail.com; 4 Mastology Program, Department of Obstetrics and Gynecology, Hospital Universitário, Universidade Federal de Goiás (UFG), Goiás 74605-050, Brazil; ribeiroufg@hotmail.com (L.R.S.); ruffojr@terra.com.br (R.F.-J.)

**Keywords:** breast cancer, PD-L1, prognosis, immunohistochemistry

## Abstract

**Simple Summary:**

The role of PD-L1 expression in breast cancer remains controversial. Therefore, we performed a systematic review and meta-analysis to assess the association of PD-L1 expression with clinicopathological variables, overall survival (OS), and disease-free survival (DFS) in invasive breast cancer. PD-L1 expression was associated with age ≥ 50 years, lymph node status-negative, progesterone receptor-negative, Ki67 ≥ 20%, and human epidermal growth factor receptor 2 (HER2)-negative. PD-L1 positivity was associated with worse OS; however, there was no significant improvement in DFS. PD-L1 positivity was significantly associated with the clinicopathological characteristics of favorable and unfavorable prognoses. However, the final clinical outcome was associated with lower OS and had no significant association with DFS.

**Abstract:**

Programmed death ligand 1 (PD-L1) has been investigated in various types of cancer; however, the role of PD-L1 expression in breast cancer remains controversial. We performed a systematic review and meta-analysis to assess the association of PD-L1 expression with clinicopathological variables, overall survival (OS), and disease-free survival (DFS) in invasive breast cancer. A total of 965 articles were included from CINAHL, Embase, PubMed, and Scopus databases. Of these, 22 studies encompassing 6468 cases of invasive breast cancer were included in the systematic review, and 15 articles were included in the meta-analysis. PD-L1 expression was associated with age ≥ 50 years, lymph node status-negative, progesterone receptor-negative, Ki67 ≥ 20%, and human epidermal growth factor receptor 2 (HER2)-negative. PD-L1 positivity was associated with worse OS (hazard ratio, HR, 2.39; 95% confidence interval, CI, 1.26–3.52; *p* =< 0.000); however, there was no significant improvement in DFS (HR 0.17; 95% CI −0.12–0.46; *p* =< 0.252). PD-L1 positivity was significantly associated with the clinicopathological characteristics of favorable and unfavorable prognoses. However, the final clinical outcome was associated with lower OS and had no significant association with DFS.

## 1. Introduction

The development of immunotherapy provides a new mechanism of action within systemic cancer therapy, as opposed to conventional treatments that lack tumor selectivity and cause adverse side effects [1,2]. These therapies use monoclonal antibodies against specific molecules that suppress the immune system, such as the programmed cell death protein (PD-1) and programmed death ligand 1 (PD-L1) [2].

The activation of the PD-1/PD-L1 pathway leads to the suppression of the T cell-mediated immune response, minimizing chronic inflammation states and controlling the emergence of autoimmune diseases. However, tumor cells can use these checkpoint pathways to inhibit cytotoxic T cells and escape the action of the immune system. When reactivated, the T cells can initiate the direct killing of tumor cells and the secretion of immunostimulatory cytokines [1,2]. In recent years, several PD-L1 inhibitors have been approved to treat malignancies including, but not limited to, melanoma, lung, kidney, and bladder cancers. Many others are also in development with the goal of being used as new cancer immuno-oncology therapies [3].

Breast cancer (BC) is one of the most prevalent malignant tumors in women with a high mortality rate [1]. However, because breast tumors usually have a low mutational load and few intratumoral lymphocytes, the advance of immunotherapy in this population is delayed [1,2]. In recent years, preclinical and clinical studies have shown that immunotherapy is a promising treatment for BC, especially triple-negative BC [2,4,5,6]. The addition of PD-1/PD-L1 inhibitors to neoadjuvant chemotherapy has increased the pathological complete response rate in patients with triple-negative tumors [4,7]. In the metastatic setting, the addition of these inhibitors has increased progression-free survival (PFS) and even median overall survival of patients who have a PD-L1-positive peritumoral immune infiltrate [2,8].

In a recent systematic review and meta-analysis, PD-L1 upregulation was associated with worse clinical outcomes in BC patients, emphasizing the significance of PD-L1 as a prognostic marker [1]. However, another systematic review showed that the role of PD-L1 expression in determining the prognosis in adjuvant and neoadjuvant chemotherapy was controversial [9]. Moreover, the immunohistochemical analysis of PD-L1 has not been standardized. Different cutoff points and cell types have been used, and the cellular region analyzed (cytoplasm or membrane) has not been defined. For instance, PD-L1 positivity was based on PD-L1 expression in tumor-infiltrating immune cells in studies with atezolizumab, in a combined score of tumor cells and immune cells in studies with pembrolizumab, and on expression in tumor cells in lung cancer studies [3,4,5].

Ongoing studies and new systematic reviews and meta-analyses are necessary to define the criteria of immunohistochemical positivity for PD-L1 and its association with the clinical course of BC. This study investigated the association of PD-L1 expression with clinicopathological characteristics, overall survival (OS), and disease-free survival (DFS) in invasive BC (IBC).

## 2. Materials and Methods

This study was reported according to Preferred Reporting Items for Systematic Reviews and Meta-Analyses (PRISMA) guidelines [10] and registered on the International Prospective Register of Systematic Reviews (PROSPERO) database (CRD42020190261). The study involved the following: (1) defining the objectives, (2) establishing the inclusion and exclusion criteria, (3) defining the information to be extracted from the articles, (4) analyzing data, (5) interpreting the results, and (6) discussing the results.

### 2.1. Search Strategy

A systematic literature search was performed using CINAHL, Embase, PubMed, and Scopus. The search terms “Breast Cancer” and “PD-L1 expression” were used as recommended by Stovgaard et al. [9] to identify the largest number of articles. The included studies were published between 1 January 2018, and 28 January 2021. The search strategy for all databases can be found in Table 1. In addition, the search strategy was supplemented by (a) citation tracking in the reference list of the included studies and relevant systematic reviews and (b) via Google Scholar searches.

### 2.2. Eligibility Criteria

The inclusion criteria were (1) observational or interventional studies on PD-L1 expression in IBC; (2) studies evaluating the prognostic ability of PD-L1 expression by immunohistochemistry; (3) studies without language restriction; and (4) articles published between 1 January 2018, and 28 January 2021. The exclusion criteria included theses, dissertations, case studies, animal studies, reviews, editorials, letters to the editor, duplicate studies, studies with specific populations (e.g., pregnant or lactating women), studies that evaluated rare histological types, and studies restricted to HER2-positive or triple-negative molecular subtypes. The full texts of the articles were requested from the authors [11]. There were no restrictions based on the treatment received for BC.

The search was limited to articles published in the period between August 2018 and January 2021 due to the existence of other systematic reviews based on prior data [1,9,11]. This study focused on discussing the latest evidence on this topic.

### 2.3. Study Selection

Titles and abstracts were screened by two researchers (M.B.C. and C.R.M.) using Rayyan software. The articles were read in full and selected according to the inclusion and exclusion criteria. Disagreements on the quality of evidence were discussed amongst the research team.

The following data were extracted: study authors and year of publication, experimental design, country, number of patients, age, sample type, data evaluation methods, and clinical outcomes (clinicopathological characteristics and survival—OS and DFS). The clinicopathological variables included age, tumor size, lymph node status, estrogen receptor (ER) status, progesterone receptor (PR) status, HER2 status, Ki-67 index, and molecular subtypes (luminal A and B, HER2-negative, and triple-negative).

### 2.4. Risk of Bias and Analysis of the Quality of Evidence

Risk of bias (RoB) was assessed in primary-level studies using the Quality in Prognosis Studies (QUIPS) tool, supported by Cochrane Prognosis Methods Group for prognosis studies [12,13]. QUIPS considers the following domains: (1) study participation, (2) study attrition, (3) prognostic factor measurement, (4) outcome measurement, (5) study confounding, and (6) statistical analysis and reporting.

The quality of the scientific evidence was evaluated using the Grading of Recommendations, Assessment, Development, and Evaluation (GRADE) online software (https://gdt.gradepro.org/app/#/, accessed on 26 September 2021) [14,15] and was classified as high, moderate, low, or very low [16].

### 2.5. Training of the Reviewers

The authors who participated in the eligibility assessments were trained regarding the study inclusion/exclusion criteria and completed a practice eligibility assessment on 50 test abstracts before starting to code articles. Moreover, the authors were also trained in performing risk of bias instruments on five articles not included in the study as well as standardized analyses using Mendeley and Rayyan software [17].

### 2.6. Statistical Analysis

A meta-analysis was conducted using the random-effects model for coded and stratified data on PD-L1 expression in the following cell types: tumor cells (TCs), immune cells (ICs), and (c) both tumor cells and immune cells (TCICs). The proportion of PD-L1 expression was determined in TCs and ICs according to clinicopathological variables, and the hazard ratios for OS and DFS were calculated. Proportion rates and hazard ratios with 95% confidence intervals (CIs) are shown as forest plots. To calculate the proportion, we use the command *metaprop*, grouping proportions which are specific to binomial data, allowing computation of exact binomial and test-based CIs. The degree of heterogeneity (I^2^) between the studies was calculated. I^2^ < 25%, I^2^ = 25–50%, and I^2^ > 50% indicated low, moderate, and high heterogeneity, respectively [18]. Publication bias was assessed using Egger’s test and funnel plots. All analyses were performed using STATA software (version 16.0; StataCorp, College Station, TX, USA).

## 3. Results

### 3.1. Identification of Studies

**Figure 1:**
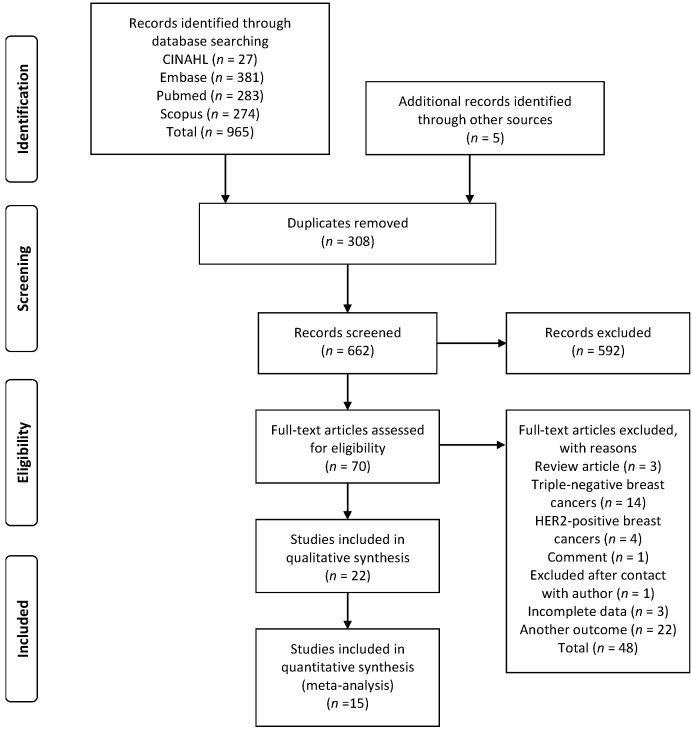
Flowchart of study selection.

A total of 965 articles were identified through database searches, and five additional studies were identified through reference lists. After removing duplicates, 662 articles were selected for reading the titles and abstracts. The decisions of the first researcher (M.B.C.) and second researcher (C.R.M.) were compared, and a Cohen kappa statistic indicated high concordance between them (93.04%; adjusted kappa, 0.80) [19]. Seventy articles were selected for full-text reading. Of these, 22 articles met the eligibility criteria and were included in the systematic review, and 15 articles were included in the meta-analysis. The flowchart of the study selection process according to the PRISMA guidelines is shown in Figure 1.

### 3.2. Study Characteristics

A total of 22 articles were included in this study [20,21,22,23,24,25,26,27,28,29,30,31,32,33,34,35,36,37,38,39,40,41]. Most of the studies were retrospective, and the follow-up interval varied from 3 months to 15 years. A total of 6468 BC cases from Italy, Greece, Japan, Korea, Germany, Egypt, the Netherlands, the United States, Sweden, and China were included in the analysis. Information on the sample type, anti-PD-L1 clones, immunohistochemical analysis criteria, and cell types is presented in Table 2. No patients received immunotherapy in the included studies.

### 3.3. PD-L1 Expression and Patient Survival

Ten studies evaluated the association between PD-L1 expression and survival in women with IBC [19,22,23,25,26,28,29,39,40,41] (Table 3). Of these, five studies determined OS [22,28,29,39,40], and five studies evaluated DFS [20,23,25,26,41]. Of these, three studies found that PD-L1 expression was associated with OS using Kaplan–Meier curves [22,39,40]. In addition, two studies investigated survival using Cox regression analysis and were included in the meta-analysis [29,40]. Four studies assessed the correlation between PD-L1 expression and DFS using Cox regression analysis and were included in the analysis [20,23,25,41] (Table 3).

### 3.4. Meta-Analysis

Of the 22 studies, 15 were included in the meta-analysis [20,21,22,23,24,26,28,29,30,32,33,37,39,40,41].

#### 3.4.1. Expression of PD-L1 in TCs, ICs, and TCICs

**Figure 2:**
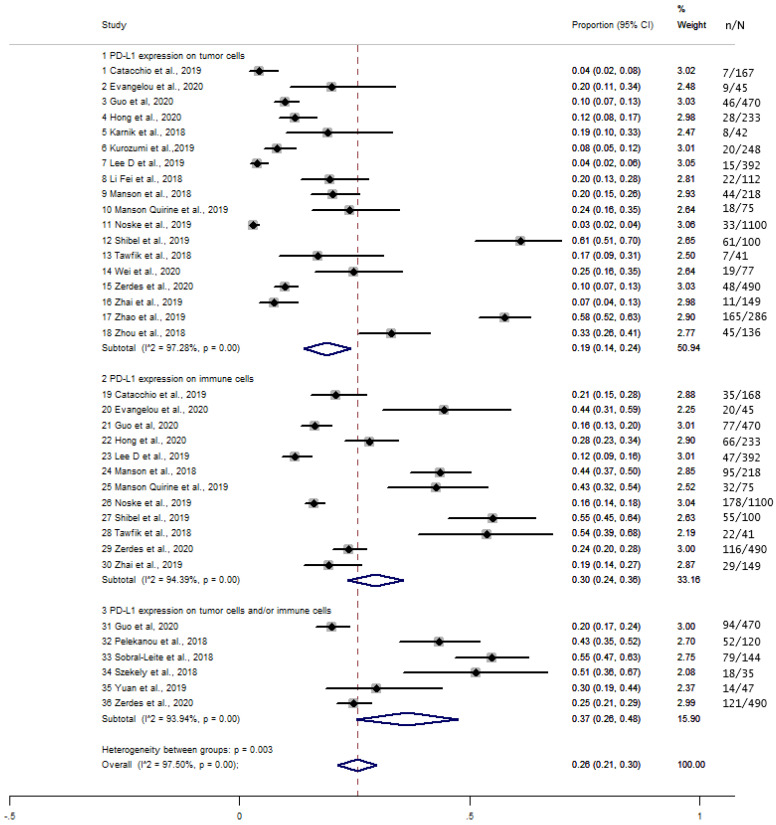
Forest plot of the proportion (%) of PD-L1 expression in tumor cells, immune cells, and both tumor and immune cells.

The overall proportion of PD-L1 expression in TCs, ICs, and TCICs was 26% (95% CI, 0.21–0.30) and was significantly higher in TCICs (37%, 30%, and 19% in TCICs, ICs, and TCs, respectively, *p* = 0.003) (Figure 2). There was significant heterogeneity between the studies (I^2^ = 97.50%, *p* < 0.001).

#### 3.4.2. PD-L1 Expression and Clinicopathological Characteristics

The proportion of PD-L1 expression in TCs and ICs was determined according to the following clinicopathological variables: age, tumor size, lymph node status, hormone receptor (ER, PR, HER2) status, Ki-67 index, and molecular subtypes (luminal A and B, HER2-positive, and triple-negative). The results were extracted from the meta-analysis graphs and are summarized in Table 4. All meta-analysis graphs that analyzed the expression of PD-L1 in TCs and in ICs according to clinical-pathological characteristics are available in Figure S1.

#### 3.4.3. Age

The proportion of PD-L1 expression was significantly higher in patients aged <50 years vs. those aged ≥50 years in TCs and ICs: 33% vs. 67% in TCs (*p* < 0.001; I^2^ = 88.86%) [17,20,36,38] and 38% vs. 62% in ICs (*p* < 0.001; I^2^ = 73.97%) [19,20,36] (Figure S1A,B). The pooled meta-analysis showed no significant difference in PD-L1 expression between TCs and ICs in women aged ≥50 years (*p* = 0.283).

#### 3.4.4. Lymph Node Status

The proportion of PD-L1 expression in ICs was higher in cases of non-lymph-node involvement (66% and 34% in node-negative and node-positive cases, respectively, *p* < 0.001), with high heterogeneity among studies (I^2^ = 84.49%, *p* < 0.001) [20,21] (Figure S1C).

#### 3.4.5. PR Status

The proportion of PD-L1 expression in TCs was significantly higher in PR-negative cases (62% vs. 38%, *p* < 0.001), and there was considerable heterogeneity between studies (I^2^ = 86.83%, *p* < 0.001) [20,21,22,23,24,28,29,32,33,40,41] (Figure S1D).

#### 3.4.6. Ki-67 Index

The frequency of PD-L1 expression in TCs was significantly higher in cases with a Ki67 index ≥ 20% (72% vs. 36%, *p* < 0.001), and there was strong heterogeneity between the studies (I^2^ = 96.48%, *p* < 0.001) [20,21,23,25,32,40,41]. Similarly, the proportion of PD-L1 expression in ICs was significantly higher in cases with a Ki67 index ≥20% (65% vs. 35%, *p* = 0.005), with high heterogeneity between the studies (I^2^ = 92.35%, *p* < 0.001) [21,23,32] (Figure S1E,F).

#### 3.4.7. HER2 Status

The frequency of PD-L1 expression in TCs was significantly higher in HER2-negative cases (76% vs. 24%, *p* = 0.000), and there was considerable heterogeneity between the studies (I^2^ = 95.34%, *p* < 0.001) [20,21,22,23,28,29,30,40,41]. Similarly, the proportion of PD-L1 expression in ICs was significantly higher in HER2-negative cases (74% vs. 26%, *p* < 0.001), with high heterogeneity among studies (I^2^ = 97.83%, *p* < 0.001) [20,21,22,23,28,29,30,39] (Figure S1G,H). The pooled meta-analysis showed no significant difference in the frequency of PD-L1 expression between TCs and ICs in HER2-negative cases (*p* = 0.283).

#### 3.4.8. PD-L1 Expression and OS

**Figure 3:**
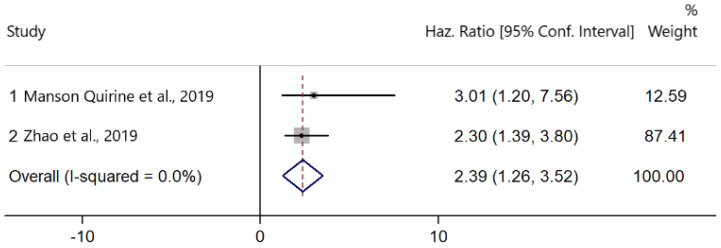
Forest plots of hazard ratios (HRs) for the effect of PD-L1 upregulation on overall survival (OS).

Two studies presented data on the association between PD-L1 upregulation and OS [28,40]. There was no heterogeneity among the studies (I^2^ = 0.0%, Cochran’s Q *p* = 0.681). PD-L1 expression was significantly associated with worse OS (HR: 2.39; 95% CI: 1.26–3.52, *p* < 0.001) (Figure 3).

#### 3.4.9. PD-L1 Expression and DFS

**Figure 4:**
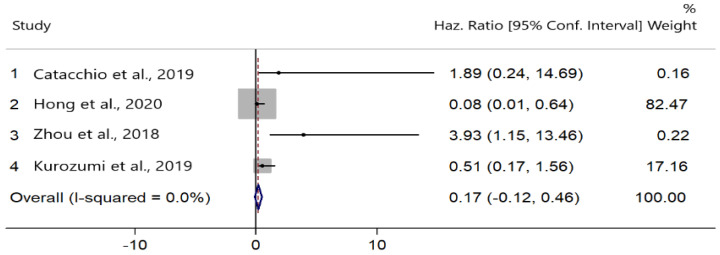
Forest plots of hazard ratios (HRs) for the effect of PD-L1 upregulation on disease-free survival (DFS).

Four studies evaluated the correlation between PD-L1 expression and DFS [20,23,25,41]. There was no heterogeneity among the studies (I^2^ = 0.0%, Cochran’s Q *p* = 0.415). PD-L1 upregulation did not significantly improve DFS (HR = 0.17; 95% CI: −0.12, 0.46; *p* = 0.252) (Figure 4).

### 3.5. Quality Assessment and Risk of Bias

The quality of scientific evidence was evaluated with the GRADE quality assessment tool, and the risk of bias was calculated using the Cochran Collaboration Risk of Bias Tool for non-randomized studies. The analysis of GRADE scores indicated that two studies had a high quality of evidence [30,38], 15 studies presented moderate quality, and four studies had low quality of evidence [21,34,36,37] (Table 1).

**Figure 5:**
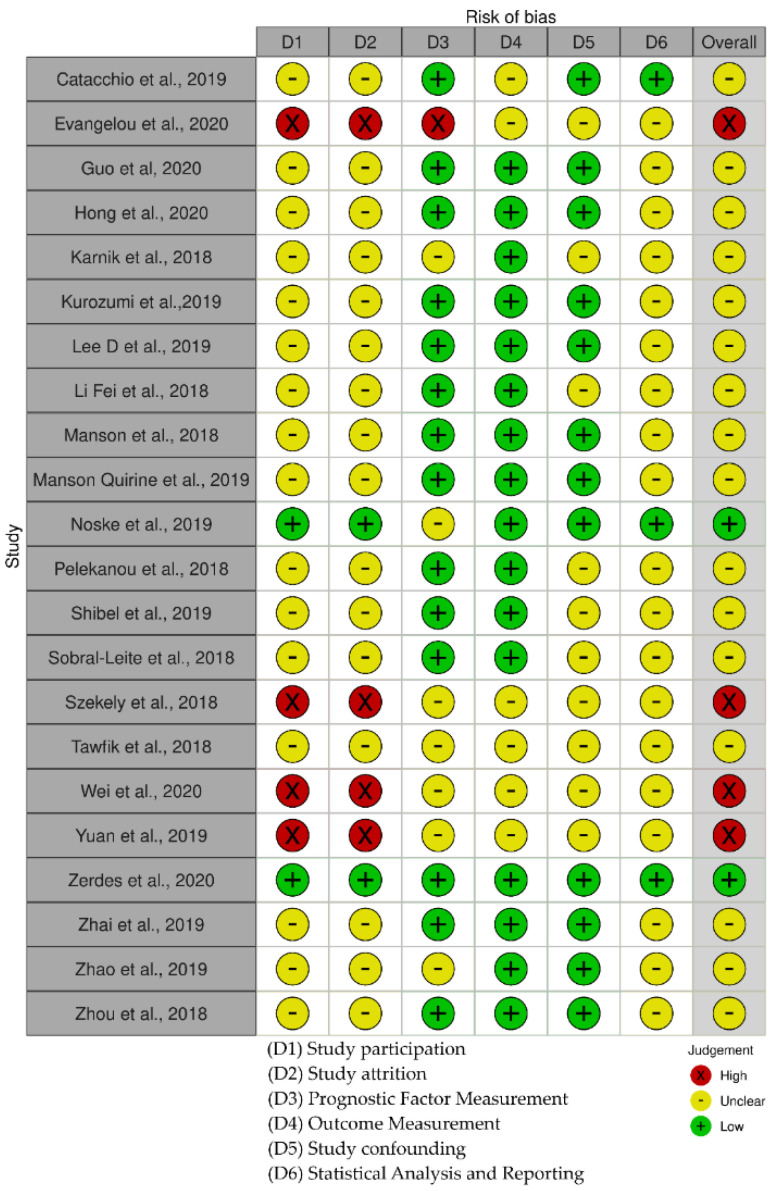
Quality plot graphically representing the risk of bias (RoB) analysis. The most relevant sources of bias were assessed in primary-level studies using the Quality in Prognosis Studies (QUIPS) tool.

The risk of bias RoB scores are shown in Figure 5. The risk of bias was moderate to low in most studies. The low quality of evidence and high risk of bias were due to small sample size and conflicting data.

### 3.6. Publication Bias

**Figure 6:**
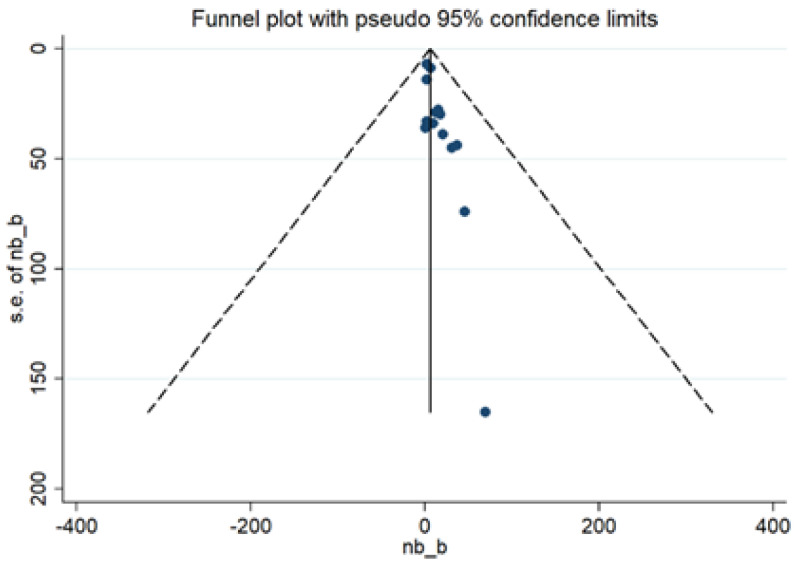
Funnel plot for the studies included in the meta-analysis.

Publication bias was evaluated using a funnel plot (Figure 6). The 15 studies included in the analysis had little publication bias. This finding was confirmed with the results from Egger’s test (*p* = 0.810).

## 4. Discussion

We identified and analyzed studies that investigated the clinicopathological features and prognostic ability of PD-L1 expression in IBC. Data from 22 studies involving 6468 BC cases were evaluated. As expected, we observed great heterogeneity between studies in relation to BC characteristics, pathologic material analyzed (TMA or full section), anti-PD-L1 clone used, determination criteria, and follow-up time. Thus, the data reported in this study will contribute to the understanding of the prognostic role of PD-L1 and its evaluation by immunohistochemistry (IHC).

The difference between the clones used and the material analyzed plays a crucial role in the rate of PD-L1 positivity, both in BCs and other tumors [42,43]. Several studies have evaluated the inter-assay variability between the different tests available for the analysis of PD-L1 expression, most with moderate correlation [22,42,44]. The SP142 assay, which predicts the response to atezolizumab in triple-negative BC, shows high interobserver agreement [45]. However, almost 30% of tumors considered PD-L1-positive by SP263 or 22C3 tests are negative by SP142 [42,45]. Regarding the analyzed pathological material, it should be noted that many of the included studies performed their analyses by TMA. Nevertheless, up to half of PD-L1 scores evaluated by TMA can be false negatives compared to whole slide evaluations [33].

The proportion of PD-L1 expression was higher in TCICs (37%), followed by ICs (30%) and TCs (19%). Another systematic review found that the frequency of PD-L1 expression was 25.8%, although the cell type studied was not reported [11]. Considering that the first indications of immunotherapy for BC may prioritize this population, defining the real proportion of PD-L1 is the first step towards the development of clinical protocols and public policies for access to medications.

Few studies have evaluated PD-L1 expression in TCICs according to their clinicopathological features. Only one study analyzed the proportion of PD-L1 expression in TCICs [22]. This study demonstrated that PD-L1 expression was significantly more prevalent in ER-positive (65.7% vs. 34.3%, *p* = 0.003), PR-negative (57.1% vs. 42.9%, *p* ≤ 0.000), and HER2-negative tumors (82.9% vs. 17.1%, *p* = 0.018) [22]. In clinical practice, the expression of TCICs has been commonly described using the combined positive score (CPS), which is the number of PD-L1 staining cells (TCs, lymphocytes, and macrophages) divided by the total number of viable TCs, multiplied by 100 [43]. In the KEYNOTE 355 study, which included cases of metastatic BC with triple-negative tumors, the addition of pembrolizumab was observed to significantly improve PFS compared with chemotherapy alone in patients with CPS ≥ 10 [46]. However, in a prognostic context, controversies remain about the most appropriate cutoff. In the present review, the studies that analyzed the expression of PD-L1 in TCICs used the proportional percentage and the 1% cutoff.

PD-L1 expression was higher in TCs and ICs in women older than 50 years, with no significant differences between the cell types. Furthermore, the frequency of PD-L1 expression was higher in ICs in patients with node-negative status. In triple-negative BC, PD-L1 positive tumors have more immunogenic characteristics, including elevated tumor infiltrating lymphocyte (TIL) and CD8 counts, enrichment of the immunogenic genomic subtype, and elevated immunogenic gene signatures at the gene expression level [47]. However, it is not clear whether these characteristics could determine the occurrence of tumors at older ages or early stages. It should be noted that the SP142 assay detects more ICs and fewer TCs compared to the other assays, which can generate conflicting results depending on the PD-L1 assay utilized [47]. Furthermore, the interobserver agreement for PD-L1 expression in ICs is inferior to TCs in various types of tumors regardless of the type of assay used, which could also contribute to the divergence of prognostic factors [3,44].

The proportion of PD-L1 expression was higher in TCs in PR-negative cases and in cases of TCs and ICs with a Ki67 index ≥20% and HER2-negative status. Another review found that PD-L1 upregulation was associated with high-risk prognostic factors, including high histological grade (*p* = 0.000), ER negativity (*p* = 0.000), PR negativity (*p* = 0.000), HER2 positivity (*p* = 0.001), and aggressive molecular subtypes (HER2-positive and triple-negative; *p* = 0.000) [11]. In parallel, a meta-analysis that included different types of epithelial-originated cancers observed an 81% increased mortality risk in a group of tumors with positive PD-L1 expression. However, the prognostic impact of PD-L1 status is more evident when stricter criteria for positive PD-L1 expression are applied [3], which reinforces the need to standardize cutoff values for each clinical setting.

In the present study, we identified that different studies were controversial in their results regarding the fact that PD-L1 expression predicts a better or worse prognosis in relation to OS. However, our meta-analysis indicated that PD-L1 expression was associated with worse OS and had no significant association with DFS, which is in agreement with two other meta-analyses [1,11]. The exact mechanism between tumor and immune microenvironment remains undetermined, but new biomarkers such as CD8 and FOXP3 may contribute to the stratification of patients and a better understanding of these survival curves [48]. Another important point involves the heterogeneity of tumor PD-L1 expression and its metastatic sites, whether in axillary lymph nodes [49] or distant metastases [28,50]. Women with PD-L1-negative primary breast tumors who developed metastatic disease with PD-L1 expression seem to improve their prognosis, which favors the inclusion of this variable in new studies [28,50]. Furthermore, although the patients included in this review did not receive any immunotherapy, the effect of the treatment received on the OS and DFS curves cannot be excluded. Finally, the standardization of PD-L1 measurement by IHC and the constant training of pathologists may, in the near future, allow new associations between the expression of checkpoint inhibitors and oncological outcomes in BC [42,43,44].

### Strengths and Limitations

This study presents the latest evidence of PD-L1 expression in IBC. In contrast to previous systematic reviews [1,11], only cases involving PD-L1 upregulation were considered. This study analyzed data from four major health science databases, and the review was conducted with scientific rigor. Moreover, studies that evaluated specific cases of BC (HER2-positive and triple-negative), which could confound the results, were not included in the analysis.

However, this study has some limitations. First, the search included only studies published in English from January 2018 to January 2021. The exclusion of non-English literature might have led to selection bias. Second, there was high heterogeneity between studies, which could be explained by differences in sample size, lack of standardized criteria for the immunohistochemical analysis of PD-L1, and the use of tissue microarrays. Future studies should use whole-tissue sections to evaluate TCICs according to clinicopathological features.

Recent data suggest that patients whose tumors overexpress PD-L1 have better clinical outcomes with immunotherapy [4,5,7,8]. In future research, intelligent clinical trials testing new combinations of immunotherapy and extensive evaluations of biomarkers are expected to be published, which could extend the current indications for immunotherapy in the management of BC. Furthermore, we suggest that further studies investigate whether the prognostic role of PD-L1 expression is different for BC patients with different therapies.

## 5. Conclusions

The proportion of PD-L1 expression was higher in TCICs. PD-L1 upregulation in TCs and ICs was associated with age ≥ 50 years, Ki67 index ≥ 20%, and tumors with lymph node-negative, PR-negative, or HER2-negative status. Moreover, PD-L1 upregulation was significantly associated with worse OS, but not with DFS.

## Figures and Tables

**Table 1:** Database search strategy.

Databases	Search Strategy
Medline/PubMed (28 January 2021)	Search: (Breast Cancer) AND (PD-L1 expression). Filters: humans, from 2018–2021, sort by: most recent ((“breast neoplasms”[MeSH Terms] OR (“breast”[All Fields] AND “neoplasms”[All Fields]) OR “breast neoplasms”[All Fields] OR (“breast”[All Fields] AND “cancer”[All Fields]) OR “breast cancer”[All Fields]) AND (“PD-L1”[All Fields] AND (“express”[All Fields] OR “expresse”[All Fields] OR “expresses”[All Fields] OR “expressing”[All Fields] OR “expressions”[All Fields] OR “gene expression”[MeSH Terms] OR (“gene”[All Fields] AND “expression”[All Fields]) OR “gene expression”[All Fields] OR “expressed”[All Fields] OR “expression”[All Fields]))) AND ((humans[Filter]) AND (2018:2021[pdat])) Total: 283
CINAHL (28 January 2021)	Boolean/phrase: breast cancer AND PD-L1 expression Limiters Published date: 2018/01/01–2021/01/28 Gender: female Total: 27
EMBASE (28 January 2021)	1 breast cancer.mp. or breast cancer/ 543437 2 programmed death 1 ligand 1/ or PD-L1 expression.mp. 32620 3 1 and 2 2698 4 limit to (human and female and yr = “2018–2021”)1029 5 limit to article Total: 381
Scopus (28 January 2021)	TITLE-ABS-KEY (breast AND cancer AND pd-l1 AND expression) AND (LIMIT-TO (PUBYEAR, 2021) OR LIMIT-TO (PUBYEAR, 2020) OR LIMIT-TO (PUBYEAR, 2019) OR LIMIT-TO (PUBYEAR, 2018)) AND (LIMIT-TO (DOCTYPE, “ar”)) AND (LIMIT-TO (EXACTKEYWORD, “Human”)) AND (LIMIT-TO (LANGUAGE, “English”)) AND (LIMIT-TO (SRCTYPE,”j”)) AND (LIMIT-TO (EXACTKEYWORD, “Female”)) Total: 274

**Table 2:** Characteristics of the studies included in the systematic review and meta-analysis.

Reference	N	Study Designs/Follow-Up (Mean)	Breast Cancer Subtype	Therapeutic Plan	Pathologic Material	Anti-PD-L1 Clone	Determination Criteria	PD-L1 Expression (TC)	PD-L1 Expression (IC)	PD-L1 Expression (TCICs)	Conflict of Interests	Ethical Approval	Quality of Evidence (GRADE)
Catacchio et al., 2019 [20] (Italy)	180	Study retrospective—cohort Follow-up: 63 months (range 3–203)	NE	CT: 16.2% (26/160) CT + hormone therapy: 40% (64/160) HT: 43.8% (70/160) Treatment data were not available: 11.1% (20/180)	TMA	SP263	*TC and IC*: only membranous staining ≥1%	7/167 (4.0%)	35/168 (21.0%)	NR	No	Yes	⊕⊕⊕◯ moderate
Evangelou et al., 2020 [21] (Greece)	45	Study retrospective—cohort Follow-up: NR	NE	NR	Full section	E1L3N	*TC*: only membranous staining ≥1% *IC*:membranous/cytoplasmic staining ≥1%	9/45 (20.0%)	20/45 (44.4%)	NR	No	NR	⊕⊕◯◯ low
Guo et al., 2020 [22] (USA)	496	Cohort Follow-up: ranged from 3 months to 154 months (median follow-up, 48 months).	ER/PR pos 73.1% (247/338) HER2 9.2% (31/338) TNBC 17.7% (60/338)	No NACT: 70.4% (349/ 496) NACT at the time of surgical excision: 29.6% (147/496)	TMA	22C3	*TC and IC:* membranous/cytoplasmic staining ≥1%	46/470 (9.8%)	77/470 (16.4%)	94/470 (20.0%)	No	Yes	⊕⊕⊕◯ moderate
Hong et al., 2020 [23] (Korea)	233	Cohort Follow-up: 45 months (1–82 months)	Luminal A 32.0% (71/222) Luminal B 41.9% (93/222) Basal 16.2% (36/222) HER2 9.9% (22/222)	CT: 85.1% (194/228) HT: 73.1% (163/223)	TMA	SP263	*TC:* membranous/cytoplasmic staining	28/233 (12.0%)	66/233 (28.3%)	NR	No	Yes	⊕⊕⊕◯ moderate
Karnik et al., 2018 [24] (USA)	136	Cohort Follow-up: NR	Luminal A: 29% (40/136) Luminal B: 40% (55/136) TN: 18% (25/136) HER2: 4% (6/136) Unknown: 7% (10/136)	NR	Full section	SP263 22C3 CAL 10	*TC:* only membranous staining ≥1%. *IC:* Not evaluated	8/42 (19.0%)	NR	NR	No	Yes	⊕⊕⊕◯ moderate
Kurozumi et al., 2019 [25] (Japan)	248	Cohort Follow-up: 128 (range, 1–147) months	HR-positive and HER2-negative: 63.7% (158/248) HER2-positive: 17.3% (43/248) Triple-negative: 19.0% (47/248)	All without CT	Full section	SP142	*TC:* cytoplasmic and/or membrane staining ≥1%. *IC*: not reported	20/248 (8.1%)	NR	NR	Yes	Yes	⊕⊕⊕◯ moderate
Lee D et al., 2019 [26] (Korea)	392	Cohort Follow-up: 89 months, 50 recurrent events occurred	Luminal A: 69.1% (271/392) Luminal B: 9.2% (36/392) HER2-positive: 8.2% (32/392) Triple-negative: 13.5% (53/392)	Adjuvant CT 77.8% (305/392) Adjuvant HT 71.9% (282/392) Adjuvant radiotherapy 66.1% (259/392). No NACT	TMA	B7-H1	*TC and IC:* not reported	15/392 (3.8%)	47/392 (12.0%)	NR	No	Yes	⊕⊕⊕◯ moderate
Li Fei et al., 2018 [27] (China)	112	Study retrospective	NR	All without radiotherapy and chemotherapy before the surgery	Full section	Abcam—polyclonal	*TC:* membranous and cytoplasmic staining	22/112 (19.6%)	NR	NR	No	NR	⊕⊕⊕◯ moderate
Manson et al., 2018 [29] (The Netherlands)	246	Cohort Follow-up: was 8.5 years (range 0.1– 22.1 years)	Luminal: 82.1% (202/246) HER2-driven: 3.7% (9/246) Triple-negative: 14.2% (35/246)	NR	TMA	SP263	*TC and IC:* membranous/cytoplasmic staining ≥1%	44/218 (20.2%)	95/218 (43.6%)	NR	No	Not required	⊕⊕⊕◯ moderate
Manson et al., 2019 [28] (The Netherlands)	106	Cohort Follow-up: 5.1 years (range 1.3–25.9 years)	Luminal: 65.7% (69/105) HER2 driven: 11.4% (12/105) Triple-negative: 22.9% (24/105)	NR	TMA	SP263	*TC and IC:* membranous/cytoplasmic staining ≥1%	18/75 (24.0%)	32/75 (42.7%)	NR	No	Not required	⊕⊕⊕◯ moderate
Noske et al., 2019 [30] (Germany)	1318	GAIN-1 study (ClinicalTRials.gov NCT0019687) was a prospective multicenter phase III trial Follow-up: NR	Luminal A: 42.0% (542/1318) Luminal B: 36.0% (465/1318) ER-/PR-/HER2+: 7.9% (102/1318) Triple-negative: 14.1% (182/1318)	Epirubicin, paclitaxel and cyclophosphamide: 50.4% (664/1318) Epirubicin, cyclophosphamide, paclitaxel and capecitabine: 49.6% (654/1318)	TMA	SP263	Cellular localization: TC: cell membrane (partially or completely stained). Cytoplasmatic staining was disregarded. IC: any PD-L1 staining (membrane/cytoplasm)	33/1100 (3.0%)	178/1100 (16.2%)	NR	Yes	Yes	⊕⊕⊕⊕ high
Pelekanou et al., 2018 [31] (USA)	211	Study prospectively—Cohort Follow-up: 3 years	NE	CT: 46,5% (98/211)	Full section	22C3	*TC and IC:* membranous/cytoplasmic staining ≥1%	NR	NR	52/120 (43%)	No	NR	⊕⊕⊕⊝ moderate
Shibel et al., 2019 [32] (Egypt)	100	Cross-sectional study Follow-up: NR	Luminal A: 32% (32/100) Luminal B: 42% (42/100) HER2 enriched: 10% (10/100) Triple-negative: 16% (16/100)	Cases who received neo-adjuvant therapy were excluded; either hormonal or chemotherapy	Full section	Polyclonal (Novus Biologicals)	*TC and IC:* membranous/cytoplasmic staining ≥1%	61/100 (61%)	55/100 (55.0%)	NR	No	Yes	⊕⊕⊕◯ moderate
Sobral-Leite et al., 2018 [33] (The Netherlands)	118	Cohort Follow-up: 10-years	NE	CT: 15.4% (25/162) Endocrine therapy: 35.8% (58/162) Radiotherapy 19.1% (31/162)	TMA and full section	E1L3N	*TC:* membranous/cytoplasmic staining ≥1% *IC*: membranous/cytoplasmic staining ≥5%	NR	NR	79/144 (54.9%)	No	Yes	⊕⊕⊕◯ moderate
Szekely et al., 2018 [34] (USA)	45	Cohort Follow-up: NR	NE	NR	TMA and full section	E1L3N	*TC and IC:* membranous/cytoplasmic staining ≥1%	NR	NR	18/35 (52.0%)	Yes	Yes	⊕⊕◯◯ low
Tawfik et al., 2018 [35] (USA)	133	Cohort Follow-up: NR	NE	NR	Full section	SP263	*TC and IC:* membranous/cytoplasmic staining ≥1%	7/41 (17.1%)	22/41 (53.7%)	NR	No	Yes	⊕⊕⊕◯ moderate
Wei et al., 2020 [36] (China)	77	Cohort Follow-up: NR	Luminal A: 11.69% (9/77) Luminal B: 61.04% (47/77) HER2-positive: 6.49% (5/77) Triple-negative: 20.78% (16/77)	Patients did not receive chemotherapy, hormone therapy or immunotherapy before surgery	Full section	EPR19759	*TC:* only membranous staining ≥ 25%. *IC*: not evaluated	19/77 (24.68%)	NR	NR	No	Yes	⊕⊕◯◯ low
Yuan et al., 2019 [37] (China)	47	Cohort Follow-up: NR	Luminal A: 21% (10/47) Luminal B: 49% (23/47) HER-2+: 21% (10/47) Triple-negative: 9% (4/47)	NR	Full section	Not reported	Not reported	NR	NR	14/47 (29.8%)	No	Yes	⊕⊕◯◯ low
Zerdes et al., 2020 [38] (Sweden)	Cohort 1 (562)Cohort 2 (1081)	Cohort Follow-up: 12.4 years and 15 years	Luminal A: 44.3% (249/562) Luminal B: 19.0% (107/562) HER2-enriched: 11.4% (64/562) Basal-like: 21.7% (122/562) Normal-like: 3.2% (18/562) Unknown: 0.4% (2/562)	ET: 29.7% (167/562) CT: 27.8% (156/562) ET/CT: 39.5% (222/562)	TMA	SP263	Not reported	48/490 (9.8%)	116/490 (23.7%)	121/490 (24.7%)	Yes	Yes	⊕⊕⊕⊕ high
Zhai et al., 2019 [39] (China)	160	Cohort Follow-up: 118 months	Luminal A: 50/160 (31.6%) Luminal B: 27.5% (44/160) Basal-like: 5.6% (9/160) Triple-negative: 23.8% (38/160)	NR	TMA	E1L3N	Not reported	11/149 (7.4%)	29/149 (19.5%)	NR	Yes	Yes	⊕⊕⊕◯ moderate
Zhao et al., 2019 [40] (China)	286	Cohort Follow-up: NR	Luminal A: 43,7% 125/286 Luminal B: 24.8% 71/286 Her2 overexpression: 11.2% 32/286 Triple-negative: 20.3% 58/286	All patients included in this study had received standardized surgery, chemotherapy, radiotherapy, endocrine therapy, and targeted therapy according to NCCN guidelines	TMA	E1L3N	TC:intensity and the percentage of cytoplasmic staining. IC: not evaluated	165/286 (57.7%)	NR	NR	No	Yes	⊕⊕⊕◯ moderate
Zhou et al., 2018 [41] (China)	136	Cohort Follow-up: 2 months and the median follow-up duration was 45.3 months	Luminal A: type 19.9% (27/136) Luminal B: type 14% (19/136) Luminal B +: type 18.4% (25/136) Her-2 Overexpression: 13.9% (19/136) Triple-negative: 33.8% (46/136)	None of the 136 patients received any form of chemotherapy, radiotherapy, endocrine therapy, or targeted therapy before surgery	Full section	Ab213524	TC: intensity and the percentage of cytoplasmic staining. IC: not evaluated	45/136 (33.1%)	NR	NR	No	Yes	⊕⊕⊕◯ moderate

NR: not reported; NE: not specified; CT: chemotherapy; HT: hormone therapy; ET: endocrine treatment; NACT: neoadjuvant chemotherapy. GRADE Working Group grades of evidence: ⊕⊕⊕⊕ high quality: Further research is very unlikely to change our confidence in the estimate of effect; ⊕⊕⊕◯ moderate quality: Further research is likely to have an important impact on our confidence in the estimate of effect and may change the estimate; ⊕⊕◯◯ low quality: Further research is very likely to have an important impact on our confidence in the estimate of effect and is likely to change the estimate; ⊕◯◯◯very low quality: We are very uncertain about the estimate.

**Table 3:** Association between PD-L1 expression and survival (overall survival and disease-free survival) in women with breast cancer.

Survival		
Overall Survival		
Reference	Follow-Up	Association—Descriptive Statistics
Guo et al., 2020 [22]	Ranged from 3 months to 154 months (median follow-up, 48 months)	Kaplan–Meier curves Positive PD-L1 staining by IC was significantly associated with worse overall survival in the subgroup with NACT (*p*= 0.021) PD-L1 staining by TCICs showed a trend for worse overall survival (*p*= 0.064)
Manson et al., 2018 [29]	8.5 years (range 0.1–22.1 years)	Kaplan–Meier curves PD-L1 *p* = 0.564 PD-L1 tumor cells (*p* = 0.776) PD-L1 immune cells (*p* = 0.83)
Manson Quirine et al., 2019 [28]	5.1 years (range 1.3–25.9 years)	Kaplan–Meier curves PD-L1 tumor cells (*p* = 0.449) Univariate Cox regression analysis HR 3.013, CI 1201–7561, *p* = 0.019
Zhai et al., 2019 [39]	118 months	Kaplan–Meier curves Tumoral or stromal PD-L1 expression were linked to better survival outcome (*p* = 0.047 and *p* = 0.026)
Zhao et al., 2019 [40]	NR	Kaplan–Meier curves Expression of PD-L1 is significantly associated with OS (*p* = 0.001) High PD-L1 expression patients had significantly shorter OS Univariate Cox regression analysis PD-L1 HR 2.299, 95% CI 1.389–3.803, *p*= 0.001
Disease-Free Survival		
Reference	Follow-Up	Association—Descriptive Statistics
Catacchio et al., 2019 [20]	63 months (range 3–203)	Univariate Cox regression analysis *TILs* HR 2.06, 95% CI 0.62–6.85, *p*= 0.228 Tumor cells HR 1.89, 95% CI 0.24–14.69, *p*= 0.534
Hong et al., 2020 [23]	45 months (1–82 months)	Univariate Cox regression analysis HR 0.084,95%, CI 0.011–0.645, *p*= 0.017
Lee D et al., 2019 [26]	89 months, 50 recurrent events occurred	Kaplan–Meier curves Expression of PD-L1 (TILs) (5-year DFS 100.0% vs. 87.7%, *p* =0.090) The estimated 5-year DFS of the entire cohort was 89.1%
Zhou et al., 2018 [41]	2 months and the median follow-up duration was 45.3 months	Multivariate Cox regression analysis PD-L1 in tumor cells was found to be an independent prognostic risk factor with the PFS rate for breast invasive ductal carcinoma, HR = 3.93, 95% CI 1.15–13.46, *p* =0.003) Kaplan–Meier curves Kaplan–Meier estimates of the progression-free survival of patients with PD-L1 expression (*p* =0.018)
Kurozumi et al., 2019 [25]	128 (range, 1–147) months	Kaplan–Meier curves of overall survival PD-L1 expression was not an independent prognostic facto (HR = 0.51, 95% CI 0.17–1.56, *p* = 0.24).

HR: hazard ratio.

**Table 4:** Proportion of PD-L1 expression in tumor cells and immune cells according to clinicopathological variables.

	PD-L1-TC	*p* Value	References	PD-L1-IC	*p* Value	References
Age (years)		<0.001	[20,23,39,41]		<0.001	[22,23,39]
<50	33%			38%		
≥50	67%			62%		
Tumor size (cm)		0.990	[20,22,23,25,27,29]		0.791	[20,22,23,29]
≤2	49%			51%		
>2	49%			49%		
Lymph node status		0.190	[20,21,27,41]		<0.001	[20,21]
(–)	42%			66%		
(+)	48%			34%		
ER		0.094	[20,21,22,23,28,29,32,33,40,41]		0.076	[20,21,22,23,28,29,32,39]
(–)	60%			44%		
(+)	47%			56%		
PR		<0.001	[20,21,22,23,24,28,29,32,33,40,41]		0.182	[20,21,22,23,28,29,32,39]
(–)	62%			56%		
(+)	38%			46%		
MIB1/ki67 expression		0.023	[20,21,23,25,32,40,41]		0.005	[20,21,23,32]
Low	36%			35%		
High	72%			65%		
HER2		<0.001	[20,21,22,23,24,28,29,30,40,41]		<0.001	[20,21,22,23,28,29,30,39]
(–)	76%			74%		
(+)	24%			26%		
Molecular subtypes						
		-	[23,26,30,32,37,40]		0.478	[23,26,30,31,39]
Luminal A	21%			16%		
		-	[23,26,32,37,39,40,41]		0.610	[23,26,32,39]
Luminal B	24%			29%		
		-	[30,32,37,40]		0.639	[30,32]
HER2 overexpression	13%			11%		
		-	[24,26,30,32,37,40]		0.751	[26,30,32,39]
TNBC	40%			37%		

I2: heterogeneity between groups; ER: estrogen receptor; RP: progesterone receptor; TNBC, triple-negative breast cancer; *p* < 0.05: statistically significant. Note: Proportion data were extracted from the meta-analysis graphs.

## Supplementary Materials

The following are available online at https://www.mdpi.com/article/10.3390/cancers13236090/s1, Figure S1: Forest plot of prevalence (%) of PD-L1 expression according to Age (A,B), Lymph node status (C), PR status (D), Ki-67 index (E,F) and HER2 status (G,H).
