# Supplementary material for: Prognostic Role of PD-L1 Expression in Invasive Breast Cancer: A Systematic Review and Meta-Analysis
Source: Cancers (Basel). 2021 Dec 3;13(23):6090. doi: 10.3390/cancers13236090 (PMC8656531; doi:10.3390/cancers13236090)
Supplement: Supplementary file 1 [file cancers-13-06090-s001.zip › cancers-1414406-supplementary.pdf]

A

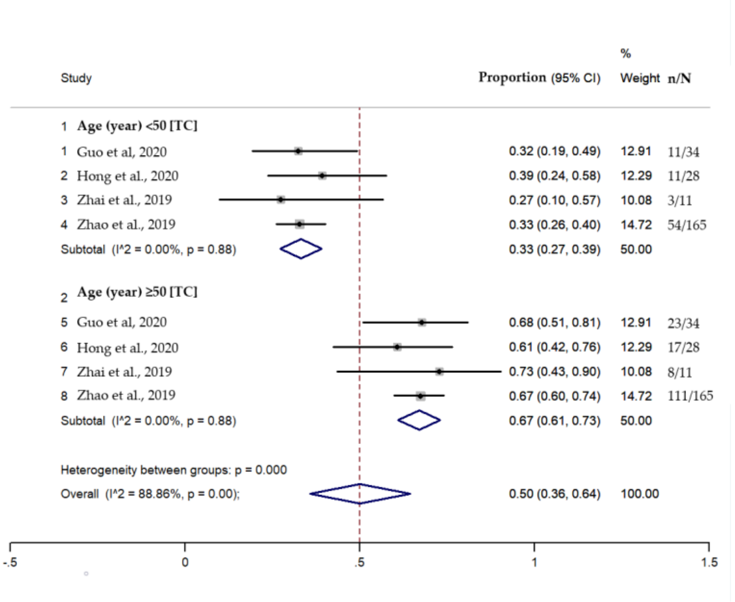

%
Study
Proportion (95% CI)
Weight
n/N
1 Age (year) <50 [TC]
1 Guo et al, 2020
0.32 (0.19, 0.49)
12.91
11/34
2 Hong et al., 2020
0.39 (0.24, 0.58)
12.29
11/28
3 Zhai et al., 2019
0.27 (0.10, 0.57)
10.08
3/11
4 Zhao et al., 2019
0.33 (0.26, 0.40)
14.72
54/165
Subtotal (I^2 = 0.00%, p = 0.88)
0.33 (0.27, 0.39)
50.00
2 Age (year) ≥50 [TC]
5 Guo et al, 2020
0.68 (0.51, 0.81)
12.91
23/34
6 Hong et al., 2020
0.61 (0.42, 0.76)
12.29
17/28
7 Zhai et al., 2019
0.73 (0.43, 0.90)
10.08
8/11
8 Zhao et al., 2019
0.67 (0.60, 0.74)
14.72
111/165
Subtotal (I^2 = 0.00%, p = 0.88)
0.67 (0.61, 0.73)
50.00
Heterogeneity between groups: p = 0.000
Overall (I^2 = 88.86%, p = 0.00);
0.50 (0.36, 0.64)
100.00
-.5
0
.5
1
1.5

B

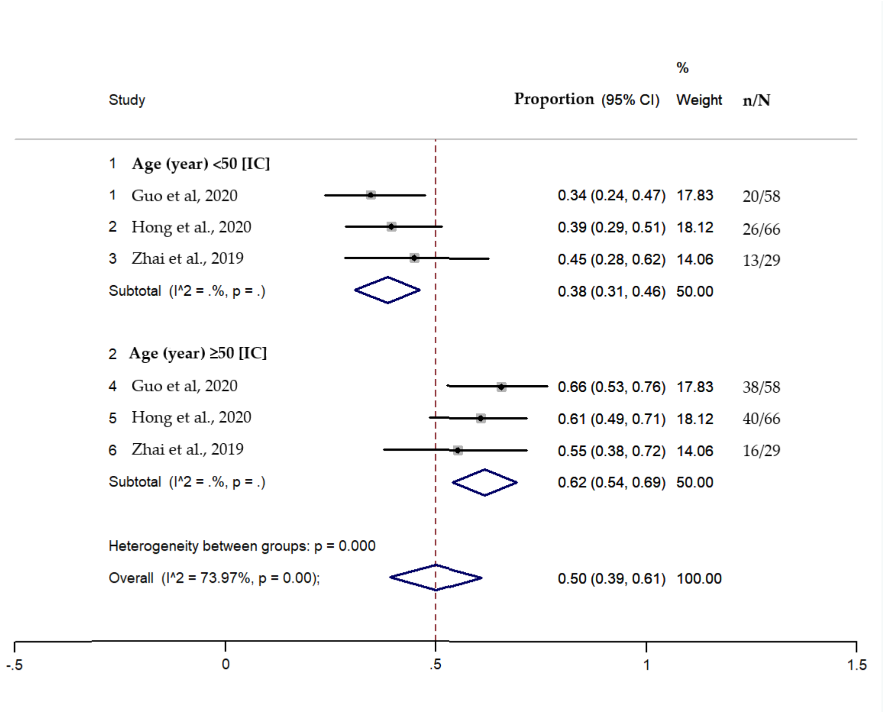

%
Study
Proportion (95% CI)
Weight
n/N
1 Age (year) <50 [IC]
1 Guo et al, 2020
0.34 (0.24, 0.47)
17.83
20/58
2 Hong et al., 2020
0.39 (0.29, 0.51)
18.12
26/66
3 Zhai et al., 2019
0.45 (0.28, 0.62)
14.06
13/29
Subtotal (I^2 = .%, p = .)
0.38 (0.31, 0.46)
50.00
2 Age (year) ≥50 [IC]
4 Guo et al, 2020
0.66 (0.53, 0.76)
17.83
38/58
5 Hong et al., 2020
0.61 (0.49, 0.71)
18.12
40/66
6 Zhai et al., 2019
0.55 (0.38, 0.72)
14.06
16/29
Subtotal (I^2 = .%, p = .)
0.62 (0.54, 0.69)
50.00
Heterogeneity between groups: p = 0.000
Overall (I^2 = 73.97%, p = 0.00);
0.50 (0.39, 0.61)
100.00
-.5
0
.5
1
1.5

C

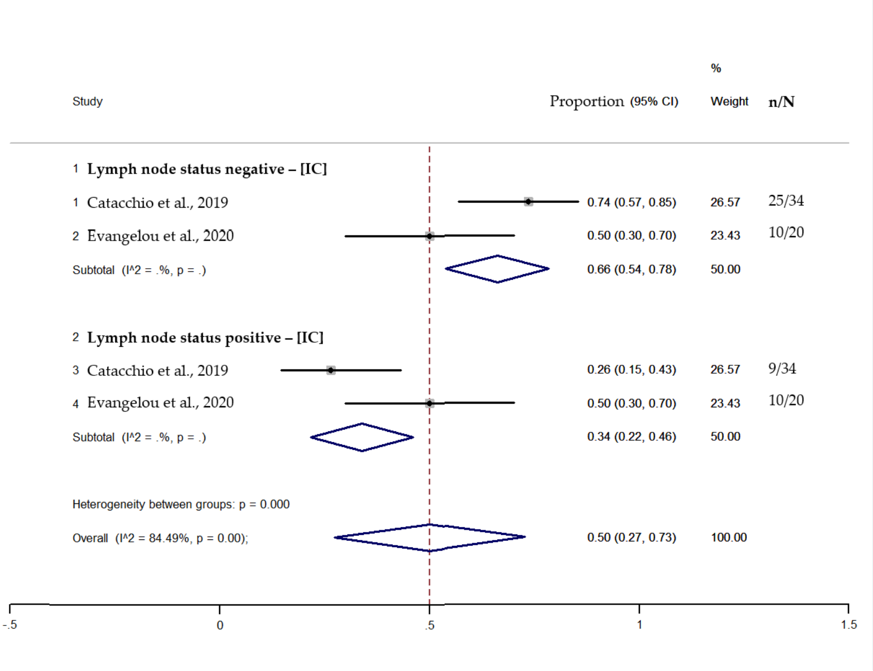

Study
Proportion (95% CI)
%
Weight
n/N
1 Lymph node status negative – [IC]
1 Catacchio et al., 2019 0.74 (0.57, 0.85) 26.57 25/34
2 Evangelou et al., 2020 0.50 (0.30, 0.70) 23.43 10/20
Subtotal (I^2 = .%, p = .) 0.66 (0.54, 0.78) 50.00
2 Lymph node status positive – [IC]
3 Catacchio et al., 2019 0.26 (0.15, 0.43) 26.57 9/34
4 Evangelou et al., 2020 0.50 (0.30, 0.70) 23.43 10/20
Subtotal (I^2 = .%, p = .) 0.34 (0.22, 0.46) 50.00
Heterogeneity between groups: p = 0.000
Overall (I^2 = 84.49%, p = 0.00); 0.50 (0.27, 0.73) 100.00
-.5
0
.5
1
1.5

D

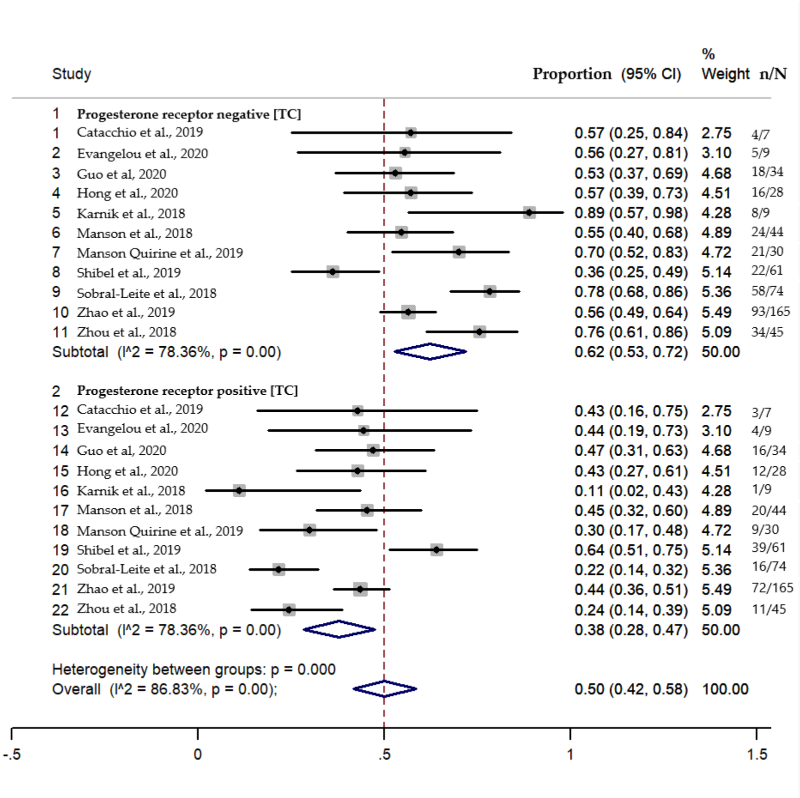

Study
Proportion (95% CI)
%
Weight n/N
1 Progesterone receptor negative [TC]
1 Catacchio et al., 2019 0.57 (0.25, 0.84) 2.75 4/7
2 Evangelou et al., 2020 0.56 (0.27, 0.81) 3.10 5/9
3 Guo et al, 2020 0.53 (0.37, 0.69) 4.68 18/34
4 Hong et al., 2020 0.57 (0.39, 0.73) 4.51 16/28
5 Karnik et al., 2018 0.89 (0.57, 0.98) 4.28 8/9
6 Manson et al., 2018 0.55 (0.40, 0.68) 4.89 24/44
7 Manson Quirine et al., 2019 0.70 (0.52, 0.83) 4.72 21/30
8 Shibel et al., 2019 0.36 (0.25, 0.49) 5.14 22/61
9 Sobral-Leite et al., 2018 0.78 (0.68, 0.86) 5.36 58/74
10 Zhao et al., 2019 0.56 (0.49, 0.64) 5.49 93/165
11 Zhou et al., 2018 0.76 (0.61, 0.86) 5.09 34/45
Subtotal (I^2 = 78.36%, p = 0.00) 0.62 (0.53, 0.72) 50.00
2 Progesterone receptor positive [TC]
12 Catacchio et al., 2019 0.43 (0.16, 0.75) 2.75 3/7
13 Evangelou et al., 2020 0.44 (0.19, 0.73) 3.10 4/9
14 Guo et al, 2020 0.47 (0.31, 0.63) 4.68 16/34
15 Hong et al., 2020 0.43 (0.27, 0.61) 4.51 12/28
16 Karnik et al., 2018 0.11 (0.02, 0.43) 4.28 1/9
17 Manson et al., 2018 0.45 (0.32, 0.60) 4.89 20/44
18 Manson Quirine et al., 2019 0.30 (0.17, 0.48) 4.72 9/30
19 Shibel et al., 2019 0.64 (0.51, 0.75) 5.14 39/61
20 Sobral-Leite et al., 2018 0.22 (0.14, 0.32) 5.36 16/74
21 Zhao et al., 2019 0.44 (0.36, 0.51) 5.49 72/165
22 Zhou et al., 2018 0.24 (0.14, 0.39) 5.09 11/45
Subtotal (I^2 = 78.36%, p = 0.00) 0.38 (0.28, 0.47) 50.00
Heterogeneity between groups: p = 0.000
Overall (I^2 = 86.83%, p = 0.00); 0.50 (0.42, 0.58) 100.00
-.5
0
.5
1
1.5

E

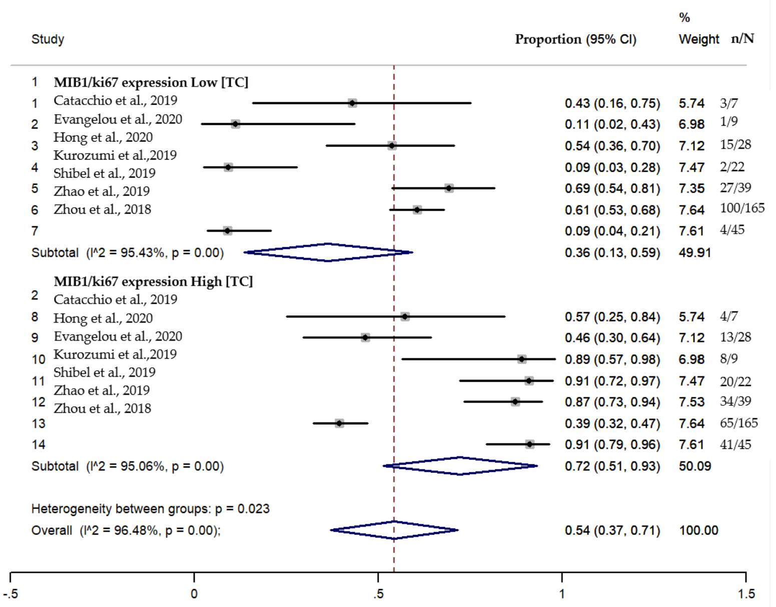
Study
Proportion (95% CI)
% Weight
n/N
1 MIB1/ki67 expression Low [TC]
1 Catacchio et al., 2019
2 Evangelou et al., 2020
3 Hong et al., 2020
Kurozumi et al.,2019
4 Shibel et al., 2019
5 Zhao et al., 2019
6 Zhou et al., 2018
7
0.43 (0.16, 0.75) 5.74 3/7
0.11 (0.02, 0.43) 6.98 1/9
0.54 (0.36, 0.70) 7.12 15/28
0.09 (0.03, 0.28) 7.47 2/22
0.69 (0.54, 0.81) 7.35 27/39
0.61 (0.53, 0.68) 7.64 100/165
0.09 (0.04, 0.21) 7.61 4/45
Subtotal (I^2 = 95.43%, p = 0.00) 0.36 (0.13, 0.59) 49.91
MIB1/ki67 expression High [TC]
2 Catacchio et al., 2019
8 Hong et al., 2020
9 Evangelou et al., 2020
10 Kurozumi et al.,2019
Shibel et al., 2019
11 Zhao et al., 2019
12 Zhou et al., 2018
13
14
0.57 (0.25, 0.84) 5.74 4/7
0.46 (0.30, 0.64) 7.12 13/28
0.89 (0.57, 0.98) 6.98 8/9
0.91 (0.72, 0.97) 7.47 20/22
0.87 (0.73, 0.94) 7.53 34/39
0.39 (0.32, 0.47) 7.64 65/165
0.91 (0.79, 0.96) 7.61 41/45
Subtotal (I^2 = 95.06%, p = 0.00) 0.72 (0.51, 0.93) 50.09
Heterogeneity between groups: p = 0.023
Overall (I^2 = 96.48%, p = 0.00); 0.54 (0.37, 0.71) 100.00
-.5
0
.5
1
1.5

F

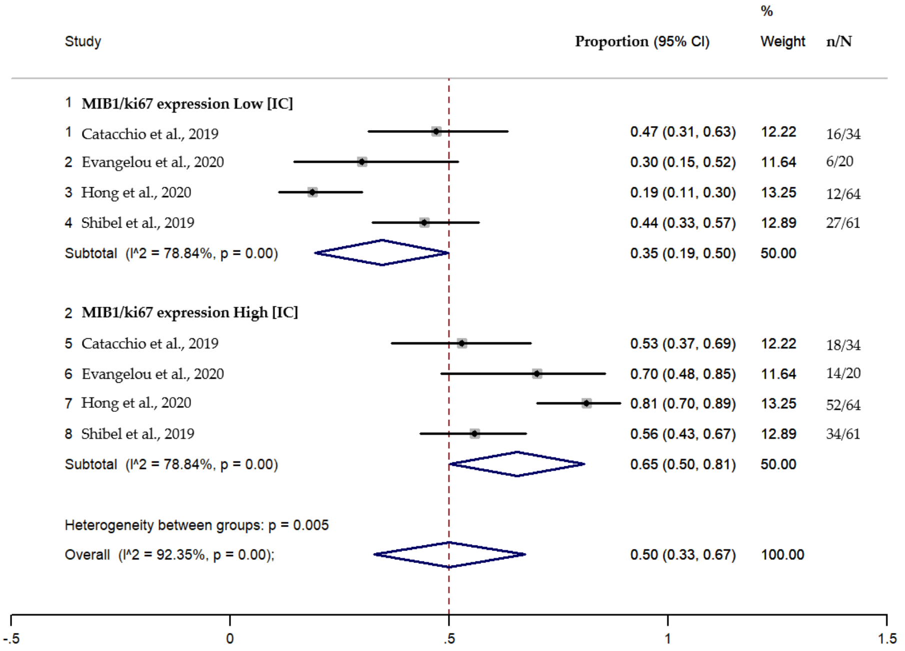
Study
Proportion (95% CI)
% Weight
n/N
1 MIB1/ki67 expression Low [IC]
1 Catacchio et al., 2019 0.47 (0.31, 0.63) 12.22 16/34
2 Evangelou et al., 2020 0.30 (0.15, 0.52) 11.64 6/20
3 Hong et al., 2020 0.19 (0.11, 0.30) 13.25 12/64
4 Shibel et al., 2019 0.44 (0.33, 0.57) 12.89 27/61
Subtotal (I^2 = 78.84%, p = 0.00) 0.35 (0.19, 0.50) 50.00
2 MIB1/ki67 expression High [IC]
5 Catacchio et al., 2019 0.53 (0.37, 0.69) 12.22 18/34
6 Evangelou et al., 2020 0.70 (0.48, 0.85) 11.64 14/20
7 Hong et al., 2020 0.81 (0.70, 0.89) 13.25 52/64
8 Shibel et al., 2019 0.56 (0.43, 0.67) 12.89 34/61
Subtotal (I^2 = 78.84%, p = 0.00) 0.65 (0.50, 0.81) 50.00
Heterogeneity between groups: p = 0.005
Overall (I^2 = 92.35%, p = 0.00); 0.50 (0.33, 0.67) 100.00
-.5
0
.5
1
1.5

G

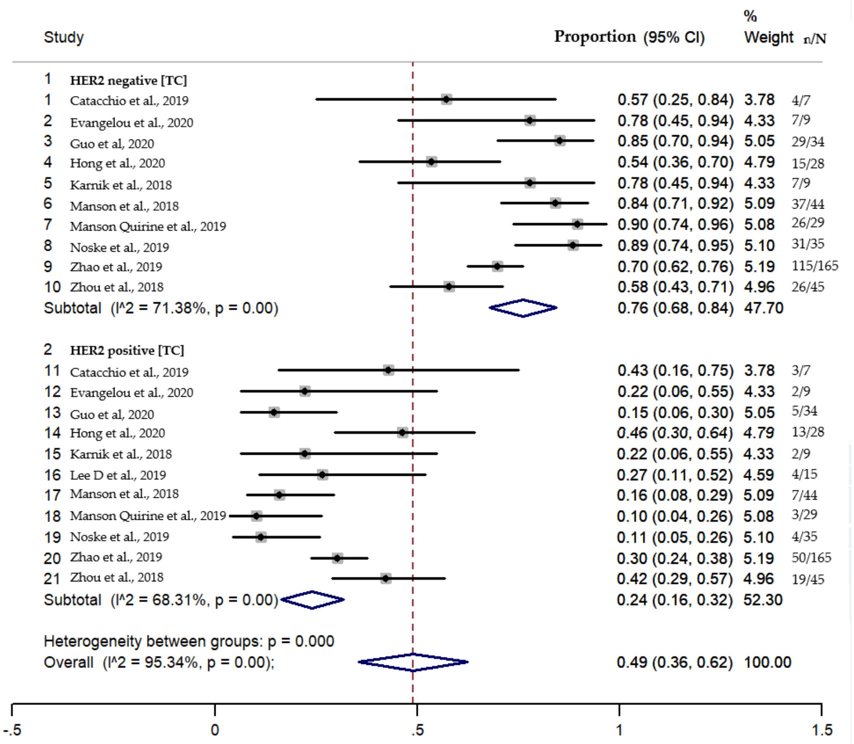

H

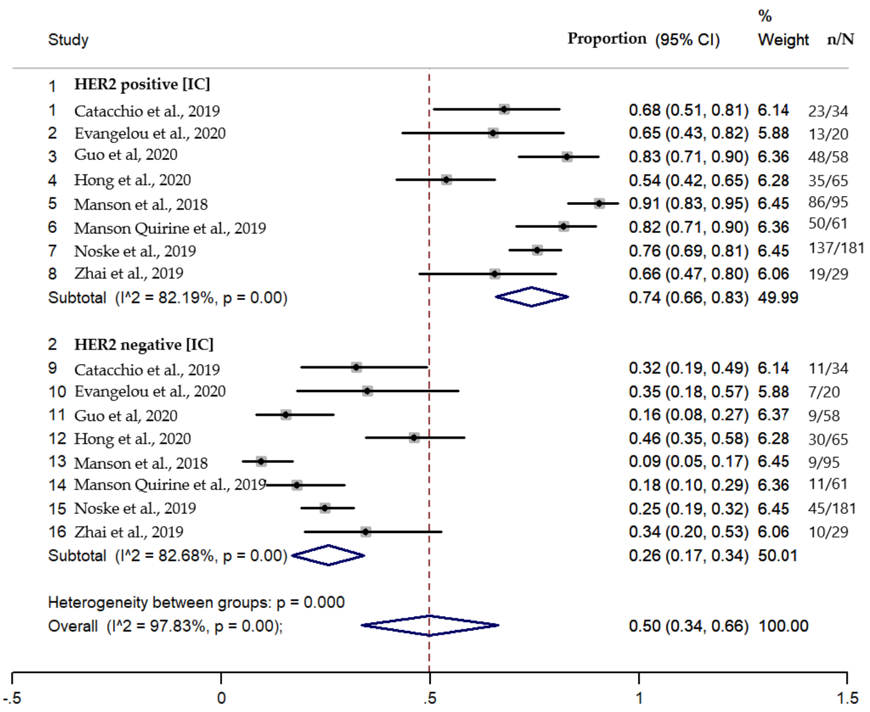

Figure S1. Forest plot of prevalence (%) of PD-L1 expression according to Age (A,B), Lymph node status (C), PR status (D), Ki-67 index (E,F) and HER2 status (G,H).
